# Peptide synthesis: ball-milling, in solution, or on solid support, what is the best strategy?

**DOI:** 10.3762/bjoc.13.206

**Published:** 2017-10-06

**Authors:** Ophélie Maurin, Pascal Verdié, Gilles Subra, Frédéric Lamaty, Jean Martinez, Thomas-Xavier Métro

**Affiliations:** 1 Institut des Biomolécules Max Mousseron (IBMM), UMR 5247, CNRS, Université de Montpellier, ENSCM, Campus Triolet, cc1703, Place Eugène Bataillon, 34095 Montpellier cedex 5, France

**Keywords:** ball-mill, green chemistry, mechanochemistry, peptide synthesis, SPPS

## Abstract

While presenting particularly interesting advantages, peptide synthesis by ball-milling was never compared to the two traditional strategies, namely peptide syntheses in solution and on solid support (solid-phase peptide synthesis, SPPS). In this study, the challenging VVIA tetrapeptide was synthesized by ball-milling, in solution, and on solid support. The three strategies were then compared in terms of yield, purity, reaction time and environmental impact. The results obtained enabled to draw some strengths and weaknesses of each strategy, and to foresee what will have to be implemented to build more efficient and sustainable peptide syntheses in the near future.

## Introduction

**Figure 1:**
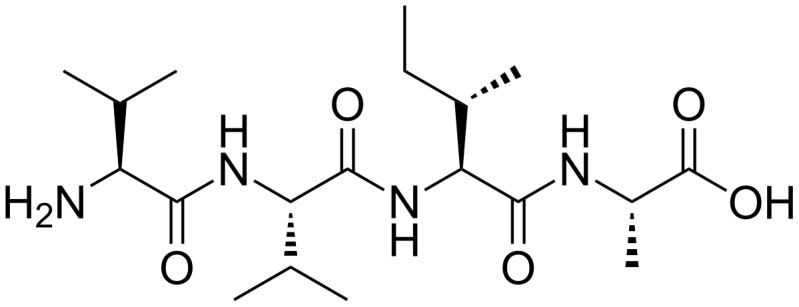
Structure of the VVIA peptide.

Peptides play a central role both in biological mechanisms and in therapeutic solutions of the future [1–2]. Pharmaceutical companies are showing a renewed interest for this type of therapeutics. A recent study showed that 140 peptides are currently evaluated in clinical trials and more than 500 are in preclinical development [3]. In the recent years, much progress has been made in the administration modes and in the strategies to improve their in vivo bioavailability and stability. This progresses empowered the potential of therapeutic peptides, suggesting a production surge in the future. Besides this high potential, actual peptide production techniques suffer from major environmental issues [4–6]. Indeed, large amounts of organic solvents (DMF, NMP, 1,4-dioxane, DCM), coupling agents (uroniums, phosphoniums, carbodiimides and auxiliary nucleophiles) and bases (Et_3_N, DIPEA, piperidine) are required for their synthesis and purification [4,7]. Unfortunately, these chemicals present highly undesirable safety profiles (flammable, corrosive and/or toxic), and industrial manufacturers are making great efforts to reduce their use [8]. All these problematic chemicals have been widely used because they furnish liquid reaction mixtures perfectly adapted to the two prevalent peptide synthesis strategies utilized in research laboratories and for industrial production: synthesis in solution and synthesis on a solid support (also known as solid-phase peptide synthesis, SPPS). Indeed, liquid reaction mixtures enable efficient agitation when using a conventional batch reactor equipped with either magnetic stirring bar or impeller, and automated handling such as pumping and filtration. Since Lamaty and co-workers have shown in their seminal work that peptide synthesis could be performed in a ball-mill (BM) [9], various solvent-free or solvent-less peptide synthesis strategies have been developed [10–17]. While these approaches enable to circumvent the use of toxic solvents and bases [18–20], no comparison between ball-milling and conventional approaches was performed, discussed and communicated. Therefore, we performed this comparison by applying three different peptide synthesis strategies (BM, solution and solid support) to the production of the VVIA peptide sequence, protected or not, depending on the strategy (all amino acids bearing L absolute configuration, Figure 1). The sequence has been chosen as it corresponds to the Aβ (39–42) tetrapeptide, a promising small therapeutic peptide that inhibits Aβ42-induced neurotoxicity [21–22], and that is known to be difficult to produce due to high hydrophobicity and steric hindrance [23].

## Results and Discussion

### Synthesis by using a ball-mill (BM)

**Scheme 1:**
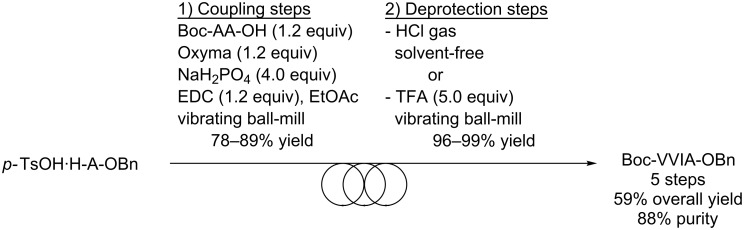
Synthesis of Boc-VVIA-OBn by the ball-milling approach.

The Boc-VVIA-OBn tetrapeptide was first synthesized by using the ball-milling strategy, based on our recent developments [14]. Thus, the coupling steps were realized by ball-milling the amino ester salts (*p*-toluenesulfonate or hydrochloride) with Boc-AA-OH (1.2 equiv) in the presence of the coupling additive ethyl cyano(hydroxyimino)acetate (also known as Oxyma, 1.2 equiv), the base NaH_2_PO_4_ (4.0 equiv) and the coupling agent *N*-(3-dimethylaminopropyl)-*N*′-ethylcarbodiimide (EDC, 1.2 equiv) in the presence of small amounts of EtOAc as the liquid grinding assistant (Scheme 1). Conventional post-treatments based on acid/base extractions and washings were sufficient to furnish the desired coupling products in satisfying purity and in isolated yields ranging from 78 to 89%. Of note, it was observed previously under similar reaction conditions that the absence of EtOAc as liquid grinding assistant (neat grinding) could lead to inhomogeneity of the reagents distribution inside the ball-mill, thereby leading to a lower overall conversion [14]. The removal of the protecting groups was performed by treatment of the Boc-protected peptides with gaseous HCl in the absence of solvents, providing the amino esters as hydrochlorides in high yield and purity (Scheme 1). Alternatively, removal of the Boc group under mechanochemical conditions was realized. While ball-milling Boc-IA-OBn with 37% aqueous HCl furnished HCl·H-IA-OBn contaminated with products arising from hydrolysis of the benzyl ester group, pure TFA·H-IA-OBn was obtained in quantitative yield by ball-milling Boc-IA-OBn with TFA (5.0 equiv) [24]. Overall, the Boc-VVIA-OBn peptide was obtained in 5 steps with 59% yield and 88% purity (Scheme 1).

### Synthesis in solution

**Scheme 2:**
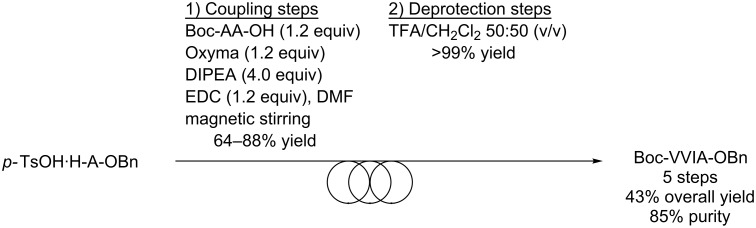
Synthesis of tetrapeptide Boc-VVIA-OBn in solution.

In parallel, the Boc-VVIA-OBn tetrapeptide was produced using the conventional synthesis in solution. For this, the amino ester salts (*p*-toluenesulfonate or hydrochloride), Boc-AA-OH (1.2 equiv), the coupling additive Oxyma (1.2 equiv) and the base *N*,*N*-diisopropylethylamine (DIPEA, 1.2 equiv) were dissolved in the minimal amount of DMF at room temperature, and then reacted with the coupling agent EDC (1.2 equiv) (Scheme 2). As described for the ball-milling approach, post-treatments based on extractions and washings furnished the desired coupling products in good purity and in isolated yields ranging from 64% to 88%. The deprotection steps were performed by dissolving the Boc-protected peptides in TFA/CH_2_Cl_2_ 50:50 (v/v) furnishing the amino esters as TFA salts in high purity and quantitative yields (Scheme 2). Overall, the Boc-VVIA-OBn peptide was obtained in 5 steps with 43% yield and 85% purity.

### Synthesis on solid support

**Scheme 3:**
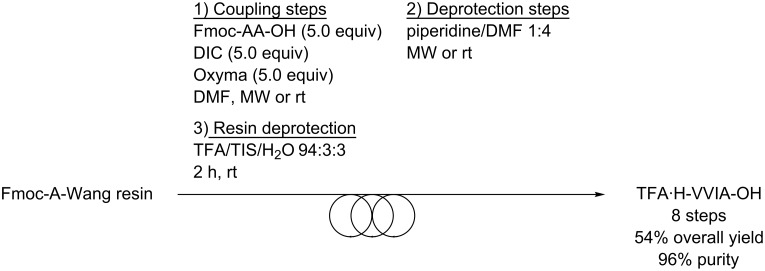
Synthesis of TFA·H-VVIA-OH by SPPS.

For the strategy involving a solid support, the chemistry was slightly different from the one used for BM or in solution, as the standard Fmoc chemistry commonly utilized in laboratories was employed [25–26]. It has to be noted that in this case the fully deprotected TFA·H-VVIA-OH peptide was obtained. Practically, the peptide chain was elongated by means of a peptide synthesizer employing the standard Fmoc chemistry (Scheme 3). The synthesis was conducted on an Fmoc-A-Wang resin on a 0.1 mmol scale with a 5-fold excess of Fmoc-protected amino acids solubilized in DMF (0.2 M), 0.5 M *N,N′*-diisopropylcarbodiimide in DMF (DIC, 5.0 equiv) as coupling reagent and 1 M Oxyma in DMF (5.0 equiv) as the coupling additive. Except for the coupling of Fmoc-V-OH with H-IA-resin and for the deprotection of Fmoc-IA-resin that were performed during 90 min at room temperature, the coupling steps were performed at 70 °C for 7 min under microwave irradiation. The deprotection steps were carried out with piperidine/DMF 1:4 for 3 min at 70 °C. After the assembly was completed, the peptide-resin was washed with CH_2_Cl_2_ and the cleavage was performed with TFA/TIS/H_2_O 94:3:3 for 2 h at room temperature. Before lyophilization, the peptide was precipitated by the addition of Et_2_O. Overall, the TFA·H-VVIA-OH peptide was obtained in 8 steps in 54% isolated yield and in 96% purity (Scheme 3).

### Comparison of the three different strategies

Having these results in hands, a comparison of the three strategies was realised. Of note, BM and solution strategies employed a Boc/Bn scenario while SPPS was based on the more conventional Fmoc/*t-*Bu scheme. Although one could point out that differences could arise from these chemical divergences, the global aim of this study was to establish a comparison based on a practical point of view. Thus, the comparison was based on the isolated yield and purity of intermediates and the final products, on the reaction time, and on the environmental impact.

#### Comparison based on the yields and purities of intermediates and final products

Contrary to SPPS where the peptide of interest is isolated at the very end of the process, syntheses performed by BM and in solution allows for a step by step comparison. Thus, for each coupling and deprotection step, the synthesis efficiency in the BM and in solution was compared based both on the isolated yields and on the purity of the peptides that were assessed by HPLC analysis (Table 1).

**Table 1:** Yields and purities for the three strategies (for each entry, the best result is indicated in bold).

		Ball-milling		Solution		SPPS	

Entry	Peptide	Yield^a^	Purity	Yield^a^	Purity	Yield^a^	Purity

1	Boc-IA-OBn	**89%**	93%	88%	**96%**	–	–
2	AH·H-IA-OBn	>99%^b^ (>99%)^c^	97%^b^ (100%)^c^	>99%^d^	99%^d^	–	–
3	Boc-VIA-OBn	**89%**	**99%**	77%	90%	–	–
4	AH·H-VIA-OBn	96%^b^	**97%**^b^	**>99%**^d^	92%^d^	–	–
5	Boc-VVIA-OBn	**78%**	**88%**	64%	85%	–	–

6	Overall	**59%**	88%	43%	85%	**54%**^e^	**96%**^e^

^a^Isolated yield. ^b^HCl salt. ^c^Obtained as TFA salt by ball-milling with 5.0 equiv TFA. ^d^TFA salt. ^e^Obtained as TFA·H-VVIA-OH.

For all coupling reactions without exception, the yields obtained under BM conditions were higher than that obtained in solution (89% vs 88% for the dipeptide, 89% vs 77% for the tripeptide and 78% vs 64% for the tetrapeptide) (Table 1, entries 1, 3 and 5). Besides, the deprotection steps always furnished the amino ester salts in excellent yields, either by using TFA/CH_2_Cl_2_ (solvent strategy) or gaseous HCl without solvent (BM strategy). On the other hand, the dipeptides were obtained with higher purity when synthesized using the conventional solution strategy compared to the BM approach (Table 1, entries 1 and 2). Yet, for all tripeptides and tetrapeptides, the BM strategy furnished the products with higher purities by 3 to 9 percentage points when compared with the solution-based approach (Table 1, entries 3–5). Overall, the 59% yield obtained with BM (Table 1, entry 6) was comparable to the one obtained with the SPPS strategy (54% yield), even more that the tetrapeptide produced by SPPS was isolated fully deprotected and with the highest purity (96%), giving additional advantage to SPPS. Yet, both in terms of overall yield and purity, the BM strategy is superior to the solution strategy (59% vs 43% overall yield and 88% vs 85% purity).

#### Comparison based on the reaction time

**Figure 2:**
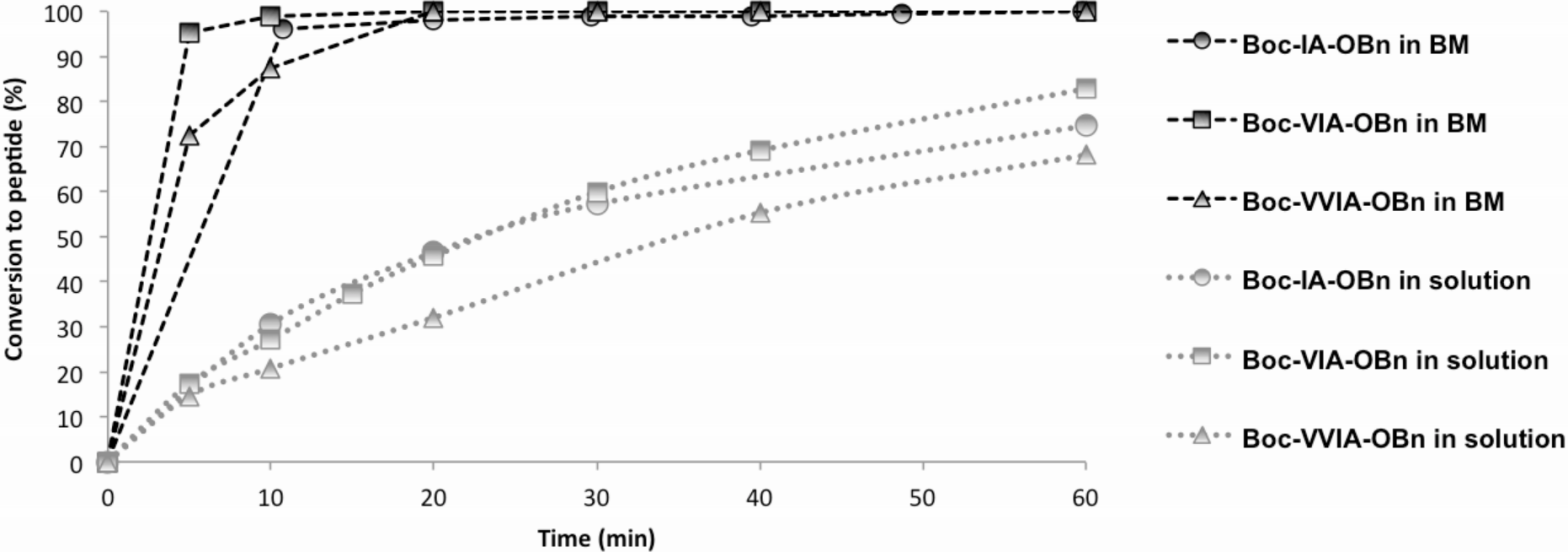
Comparison of the reaction time of the coupling steps performed in the BM and in solution.

During the course of the coupling reactions performed in the BM and in solution, aliquots were regularly sampled, quenched and analyzed by HPLC to determine the conversion. Considering the coupling steps realized in solution, the reaction mixture was dissolved in the minimal amount of DMF to ensure maximal speed of reaction while securing proper agitation. On the contrary to the solution synthesis, aliquots sampling from the milling jars implied stopping the milling process for 1–2 minutes. As one could suggest that coupling reactions could be continuing even without milling [27–30], these short pauses were considered as reaction time. As a consequence, the effective milling time was shorter than the reaction time (see Supporting Information File 1 for details). All conversions values were plotted against reaction time and the results are shown in Figure 2 below.

After 20 minutes reaction, conversions were >98% for all three coupling steps performed in the BM (Figure 2). Conversely, after the same time, none of the reaction steps done in solution reached 50% conversion. On average, coupling reactions in solution required 3 hours to reach >98% conversion, which is nine times longer than when using the BM (see Supporting Information File 1 for details). Considering the reaction time, coupling steps were considerably more efficient in BM than in solution. Whereas aliquots could be easily taken from reaction mixtures of the coupling steps, no reproducible samples could be taken for the deprotection steps using gaseous HCl. Therefore, for the deprotection no comparison of the reaction times between the two strategies (BM and solution) was possible. Similarly, the speed of reaction was not measured for SPPS, as automation of the coupling and deprotection steps enabled to save a considerable amount of time compared to ball-milling and conventional synthesis in solution. Indeed, post-treatments in BM and in solution strategies were performed by hand. Thus, a few days were necessary to complete the synthesis when using the BM or solution strategies. For comparison, half a day was sufficient to produce the VVIA sequence when using SPPS.

#### Comparison based on the environmental impact

Finally, the three different strategies were compared in terms of environmental impact. The widely used E-factor [31–33], which is defined as follows:


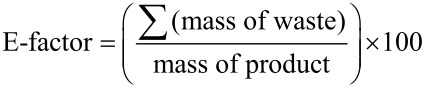


was calculated for the coupling and deprotection steps of each strategy (Table 2). Of note, the amount of reactants used in all three strategies were either based on previously optimized reaction conditions or reduced as much as possible without hampering the success of the reaction. This was realized to ensure relevant comparison between the different strategies. For all coupling steps, the E-factor obtained with the BM strategy outperformed the two other strategies, SPPS producing from seven to twenty times more waste than the BM strategy (Table 2, entries 1, 3 and 4). Unfortunately, the experimental set-up for the removal of the Boc protection group with gaseous HCl prevented the measurement and the optimization of gaseous HCl quantities required complete the reaction. Consequently, the E-factor corresponding to the Boc deprotection steps using gaseous HCl could not be calculated. Yet, deprotection of Boc-IA-OBn by ball-milling in the presence of TFA enabled to calculate the E-factor, which was five times less than in solution and more than thousand times less than SPPS (Table 2, entry 2).

**Table 2:** Comparison of the E-factor between the three strategies (for each entry, the best result is indicated in bold).

		E-factor		
		
Entry	Peptide	Ball-milling	Solution	SPPS

1	P-IA-OR	**4.9**	7.3	95.5
2	TFA^.^H-IA-OR	**1.3**	5.9	1406.6
3	P-VIA-OR	**5.0**	7.1	81.0
4	P-VVIA-OR	**9.4**	17.8	68.1

While providing an interesting insight into the amount of waste produced in each strategy, the E-factor does not provide any information concerning the toxicity of the reactants used. Pursuing an initiative we started previously [34], we calculated the cumulative Number of the Hazard Phrases (cNHP) indicated in the safety data sheets (SDS) of the reactants used in each strategy (Table 3).

**Table 3:** Comparison of the cumulative Number of Hazard Phrases (for each entry, the best result is indicated in bold).

		Cumulative Number of Hazard Phrases		
		
Entry	Reaction	Ball-milling	Solution	SPPS

1	coupling	**4**	11	12
2	deprotection with HCl(g)	**3**	–	–
3	deprotection with TFA	**3**	9	–
4	deprotection with Pip/DMF	–	–	11

As expected, the ball-milling strategy was the one for which this number was the lowest for each of the coupling and deprotection steps, corresponding to the safest approach in terms of toxicity. Of note, various research groups have screened greener solvents for SPPS [35–38]. The results issuing from these studies indicate that a reduction of the cumulative Number of Hazard Phrases in both the coupling and deprotection steps may be accessible by choosing more appropriate solvents than DMF. While this is highly positive information for the development of greener peptide syntheses, these strategies are yet inefficient in reducing the total amount of waste, which is one of the main drawbacks of SPPS.

## Conclusion

Overall, both in terms of yield and purity, the efficiency of the three strategies can be ranked as follows: BM ≈ SPPS > solution. Of note, the solution strategy gave the dipeptides with higher purity than the ball-milling approach. Although SPPS is the strategy of choice towards long peptides so far, this study showed that ball-milling was superior to the solution synthesis when considering long peptides. Similarly, ball-milling proved far more efficient than the synthesis in solution when considering the reaction time of the coupling steps. Although producing the peptide of interest with the highest purity, SPPS also presents by far the worst environmental impact. The production of waste can range from seven to thousand times more than BM. Regarding the environmental impact, the three strategies can be ranked as follows: BM > solution >> SPPS. With the increasing implementation of REACH regulations [39], one can easily foresee that the extremely low environmental impact of BM will be a determining advantage in the future. Time and money saved by automation of coupling and deprotection steps in SPPS could be transformed into a crippling burden when considering costs and environmental impact related to the use of large excesses of chemicals associated with SPPS. While SPPS has benefited from more than 50 years of research and development, and is still the method of choice for very long peptides, peptide synthesis by ball-milling is still in its infancy. Further optimization of the deprotection steps, demonstration of the feasibility to synthesize longer peptides, as well as automation of the coupling and deprotection steps would undoubtedly bring peptide synthesis by ball-milling to be the method of choice for peptide synthesis in laboratories, as well as for industrial production.

## Supporting Information

File 1 Experimental procedures and characterization data of peptides.
