# Supplementary material for: Peptide synthesis: ball-milling, in solution, or on solid support, what is the best strategy?
Source: Beilstein J Org Chem. 2017 Oct 6;13:2087–93. doi: 10.3762/bjoc.13.206 (PMC7874854; doi:10.3762/bjoc.13.206)
Supplement: File 1 — Experimental procedures and characterization data of peptides. [file Beilstein_J_Org_Chem-13-2087-s001.pdf]

Supporting Information

for

Peptide synthesis: ball-milling, in solution, or on solid support, what is the best strategy?

Ophélie Maurin, Pascal Verdié, Gilles Subra, Frédéric Lamaty[§]*, Jean Martinez and Thomas-Xavier Métro[§]*

Address: Institut des Biomolécules Max Mousseron (IBMM), UMR 5247, CNRS, Université de Montpellier, ENSCM, Campus Triolet, cc1703, Place Eugène Bataillon, 34095 Montpellier cedex 5, France

Email: frederic.lamaty@umontpellier.fr; thomas-xavier.metro@umontpellier.fr

*Corresponding author

[§]http://www.greenchem.univ-montp2.fr

Experimental procedures and characterization data of peptides

General information

Reagents were purchased from Sigma Aldrich, Fluka, Propeptide, Novabiochem or Senn Chemicals and used without further purification. All the amino acid derivatives employed in this study displayed L absolute configuration. The milling treatments were carried out in a Retsch Mixer Mill 200. SPPS was performed at the "SynBio3 Platform" (IBMM, Université de Montpellier) using a Liberty Blue peptide synthesizer (CEM). Analyses were performed at the "Plateforme Technologique Laboratoire de Mesures Physiques" (IBMM, Université de Montpellier). ^{1}H NMR spectra were recorded either on a Bruker Avance I 300 MHz or on a Bruker Avance III HD 400 MHz spectrometer and are reported in ppm using residual solvent as an internal standard ($CDCl_3$ at 7.26 ppm, DMSO-d_6 at 2.50 ppm, CD_3OD at 3.31 ppm and D_2O at 4.79 ppm). Data are reported as s = singlet, d = doublet, t = triplet, q = quadruplet, qt = quintuplet, m = multiplet; coupling constant in Hz; integration. ^{13}C NMR spectra were

recorded either on a Bruker Avance I 75 MHz or on a Bruker Avance III HD 101 MHz and are reported in ppm using residual solvent as an internal standard ($CDCl_3$ at 77.16 ppm, DMSO-d_6 at 39.52 ppm and CD_3OD at 49.00 ppm). Mass spectra were obtained by LC–ESIMS using a Waters Alliance 2695 as LC, coupled to a Waters ZQ spectrometer with electrospray source, a simple quadrupole analyzer and a UV Waters 2489 detector. Purity of products was determined by HPLC (Agilent technologies 1220 infinity LC) using Ony Monolithic HD–C18 (50 × 4.6 mm) column and a UV detector (λ = 214 nm); samples were injected with a volume of 5 µL. The solvent system used water (0.1% TFA) and acetonitrile (0.1% TFA) as eluent with a flow of 3mL/min (0–100% in 3 min).

Operating procedures and analytical data

Boc-Ile-Ala-OBn [70691-54-2][1]

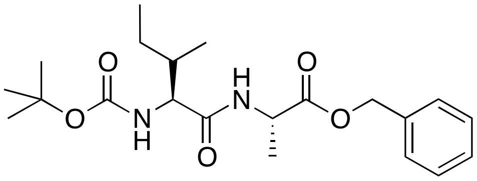

Synthesis in solution

p-TsOH·H-Ala-OBn (239.9 mg, 0.683 mmol, 1.0 equiv), Boc-Ile-OH (189.5 mg, 0.819 mmol, 1.2 equiv), Oxyma (116.2 mg, 0.818 mmol, 1.2 equiv) and *N*,*N*-diisopropylethylamine (475 µL, 2.727 mmol, 4.0 equiv) were introduced in a round-bottomed flask and solubilized in a minimum amount of DMF (1 mL) under magnetic stirring. After a few minutes of stirring, *N*-(3-dimethylaminopropyl)-*N*′-ethylcarbodiimide (145 µL, 0.819 mmol, 1.2 equiv) was added. Aliquots of the reaction mixture were withdrawn periodically and immediately quenched with a 1:1 MeCN/1 N aqueous HCl solution. Conversion was determined by HPLC analysis.

Table of conversion

Time (min)	0.0	10.0	20.0	30.0	60.0	120.0	180.0
Conversion in solution (%)	0.0	30.6	46.5	57.2	74.6	90.6	97.6

After completion of the reaction, the mixture was concentrated under vacuum and EtOAc (20 mL) and deionized water (15 mL) were added. The aqueous phase was extracted with EtOAc (2 × 10 mL) and the combined organic phase was washed with 1 N aqueous HCl solution (2 × 10 mL), 1 N aqueous NaOH solution (2 × 10 mL) and saturated aqueous NaCl solution (2 × 10 mL), dried over $MgSO_4$, filtered and concentrated under vacuum to furnish the desired product as a pale yellow solid (236.5 mg, 88% yield, 96% HPLC purity).

Synthesis in ball-mill

p-TsOH·H-Ala-OBn (239.8 mg, 0.682 mmol, 1.0 equiv), Boc-Ile-OH (189.6 mg, 0.820 mmol, 1.2 equiv), Oxyma (116.5 mg, 0.820 mmol, 1.2 equiv), NaH_2PO_4 (327.9 mg, 2.733 mmol, 4.0 equiv) and EtOAc (450 µL, η = 0.45 µL/mg) were introduced in a 15 mL PTFE grinding jar with one stainless steel ball (10 mm diameter). The jar was subjected to grinding for 2 min in the mixer mill operated at 25 Hz. *N*-(3-Dimethylaminopropyl)-*N*′-ethylcarbodiimide (145 µL,

0.819 mmol, 1.2 equiv) was added and the jar was subjected to grinding for 8 min in the mixer mill operated at 25 Hz. A first aliquot of the reaction mixture was withdrawn, immediately quenched with 1:1 MeCN/1 N aqueous HCl solution and analyzed by HPLC to determine the conversion. The jar was subjected to further grinding in the mixer mill operated at 25 Hz until next aliquot was withdrawn. This operation was repeated until completion of the reaction.

Table of conversion

Time (min)	0.0	10.8	20.0	29.6	39.5	48.8	59.8
Effective milling time (min)	0.0	8.0	16.0	24.0	32.0	40.0	48.0
Conversion in ball-mill (%)	0.0	96.2	98.1	98.8	98.9	99.4	100.0

The reaction mixture was treated with EtOAc (20 mL) and deionized water (20 mL). The aqueous phase was extracted with EtOAc (2 × 10 mL) and the combined organic phase was washed with 1 N aqueous HCl solution (2 × 10 mL), 1 N aqueous NaOH solution (2 × 10 mL) and saturated aqueous NaCl solution (2 × 10 mL), dried over $MgSO_4$, filtered, concentrated under vacuum to furnish the desired product as a white solid (238.8 mg, 89% yield, 93% HPLC purity).

Characterization

^{1}H NMR (300 MHz, $CDCl_3$): δ 7.41 – 7.28 (5H), 6.42 (br d, *J* = 6.3 Hz, 1H), 5.20 (d, *J* = 12.3 Hz, 1H), 5.14 (d, *J* = 12.0 Hz, 1H), 5.05 (br d, *J* = 7.8 Hz, 1H), 4.63 (qt, *J* = 7.2 Hz, 1H), 3.95 (br t, *J* = 7.5 Hz, 1H), 1.86 (m, 1H), 1.56 – 1.33 (13H), 1.12 (m, 1H), 0.97 – 0.82 (6H).
^{13}C NMR (101 MHz, $CDCl_3$): δ 172.6, 171.3, 155.9, 135.4, 128.7, 128.6, 128.3, 80.0, 67.3, 59.2, 48.2, 37.5, 28.4, 24.9, 18.4, 15.6, 11.5.
MS (ESI): m/z 393.2 $[M+H]^+$

TFA·H-Ile-Ala-OBn

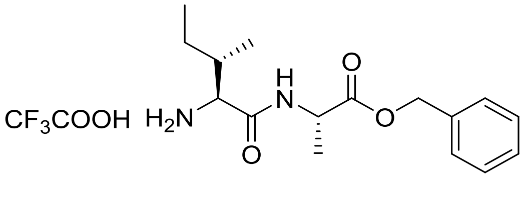

Synthesis in solution

Boc-Ile-Ala-OBn (213.5 mg, 0.544 mmol, 1.0 equiv) was solubilized in a 1:1 TFA/CH_2Cl_2 mixture (1 mL) in a round-bottomed flask with magnetic stirring. After 60 min agitation, the reaction mixture was concentrated under vacuum to furnish the desired product as a yellow solid (234.3 mg, >99% yield, 99% HPLC purity).

Synthesis in ball-mill

Boc-Ile-Ala-OBn (672.0 mg, 1.712 mmol, 1.0 equiv) and trifluoroacetic acid (TFA, 655 µL, 5.0 equiv) were introduced in a 15 mL PTFE grinding jar equipped with one PTFE ball (10 mm diameter). The jar was subjected to grinding for 4 h in the mixer mill operated at 25 Hz. The reaction mixture was recovered with CH_2Cl_2 and concentrated under vacuum to furnish the desired product as a pale yellow solid (703.2 mg, >99% yield, 100% HPLC purity).

Characterization

^{1}H NMR (300 MHz, CD_3OD): δ 7.40 – 7.27 (5H), 5.19 (d, *J* = 12.3 Hz, 1H), 5.13 (d, *J* = 12.3 Hz, 1H), 4.54 (q, *J* = 7.3 Hz, 1H), 3.71 (d, *J* = 5.6 Hz, 1H), 1.90 (m, 1H), 1.57 (m, 1H), 1.43 (d, *J* = 7.3 Hz, 3H), 1.31 – 1.10 (1H), 0.99 (d, *J* = 6.9 Hz, 3H), 0.91 (t, *J* = 7.4 Hz, 3H).
^{13}C NMR (75 MHz, CD_3OD): δ 173.4, 169.3, 137.1, 129.6, 129.4, 68.1, 58.9, 38.0, 25.3, 17.2, 14.8, 11.6.
MS (ESI): m/z 292.9 $[M+H]^+$

HCl·H-Ile-Ala-OBn [178313-32-1][2]

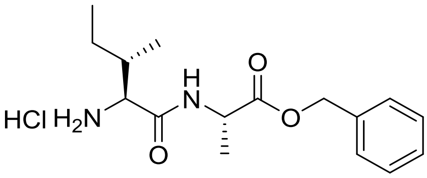

Synthesis in ball-mill

Boc-Ile-Ala-OBn (210.8 mg, 0.537 mmol, 1.0 equiv) was submitted to gaseous HCl for 3.5 h. HCl·H-Ile-Ala-OBn was recovered as a white solid (181.1 mg, >99% yield, 97% HPLC purity).

Characterization

^{1}H NMR (300 MHz, D_2O): δ 7.49 – 7.38 (5H), 5.20 (s, 2H), 4.53 (q, *J* = 7.3 Hz, 1H), 3.86 (d, *J* = 5.6 Hz, 1H), 2.01 – 1.83 (1H), 1.91 (m, 1H), 1.57 – 1.48 (1H), 1.45 (d, *J* = 7.1 Hz, 3H), 1.17 (m, 1H), 0.92 (d, *J* = 6.9 Hz, 3H), 0.85 (t, *J* = 7.4 Hz, 3H).
^{13}C NMR (101 MHz, D_2O): δ 173.7, 168.9, 135.0, 128.82, 128.77, 128.5, 67.8, 57.5, 49.0, 36.3, 24.0, 15.8, 13.6, 10.5.
MS (ESI): m/z 293.3 $[M+H]^+$

Boc-Val-Ile-Ala-OBn

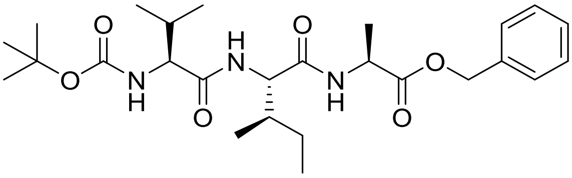

Synthesis in solution

TFA·H-Ile-Ala-OBn (178.1 mg, 0.438 mmol, 1.0 equiv), Boc-Val-OH (114.7 mg, 0.528 mmol, 1.2 equiv), Oxyma (74.9 mg, 0.527 mmol, 1.2 equiv) and *N*,*N*-diisopropylethylamine (305 µL, 1.751 mmol, 4.0 equiv) were introduced in a round-bottomed flask and solubilized in a minimum of DMF (700 µL) with magnetic stirring. After a few minutes, *N*-(3-dimethylaminopropyl)-*N′*-ethylcarbodiimide (93.1 µL, 0.526 mmol, 1.2 equiv) was added. Aliquots of the reaction mixture were withdrawn periodically and immediately quenched with 1:1 MeCN/1 N aqueous HCl solution. The conversion was determined by HPLC analysis.

Table of conversion

Time (min)	0.0	5.0	10.0	15.0	20.0	30.0	40.0	60.0
Conversion in solution (%)	0.0	17.4	27.1	37.3	45.6	59.9	69.1	82.8

After stirring for 15 h, the reaction mixture was concentrated under vacuum and EtOAc (20 mL) and deionized water (20 mL) added. The aqueous phase was extracted with EtOAc (2 × 10 mL) and the combined organic phase was washed with 1 N aqueous HCl solution (2 × 10 mL), 1 N aqueous NaOH solution (2 × 10 mL) and saturated aqueous NaCl solution (2 × 10 mL), dried over $MgSO_4$, filtered and concentrated under vacuum to furnish the desired product as a pale white solid (165.8 mg, 77% yield, 90% HPLC purity).

Synthesis in ball-mill

HCl·H-Ile-Ala-OBn (111.3 mg, 0.338 mmol, 1.0 equiv), Boc-Val-OH (88.8 mg, 0.409 mmol, 1.2 equiv), Oxyma (57.9 mg, 0.407 mmol, 1.2 equiv), NaH_2PO_4 (162.4 mg, 1.354 mmol, 4.0 equiv) and EtOAc (450 µL, η = 0.93 µL/mg) were introduced in a 15 mL PTFE grinding jar equipped with one stainless steel ball (10 mm diameter). The jar was subjected to grinding for 2 min in the mixer mill operated at 25 Hz. Then, *N*-(3-dimethylaminopropyl)-*N′*-ethylcarbodiimide (71.9 µL, 0.406 mmol, 1.2 equiv) was added and the jar was subjected to grinding for 3 min in the mixer mill operated at 25 Hz. A first aliquot of the reaction mixture was withdrawn, immediately quenched with 1:1 MeCN/1 N aqueous HCl solution and analyzed by HPLC to determine the conversion. The jar was subjected to further grinding in the mixer mill operated at 25 Hz until next aliquot was withdrawn. This operation was repeated until completion of the reaction.

Table of conversion

Time (min)	0.0	5.0	10.0	20.0	30.0	40.0	60.0
Effective milling time (min)	0.0	3.0	6.0	14.0	22.0	30.0	50.0
Conversion in ball-mill	0.0	95.3	98.8	100.0	100.0	100.0	100.0

To the reaction mixture was added EtOAc (25 mL) and deionized water (20 mL). The aqueous phase was extracted with EtOAc (2 × 10 mL) and the combined organic phase was washed with 1 N aqueous HCl solution (2 × 10 mL), 1 N aqueous NaOH solution (2 × 10 mL) and saturated aqueous NaCl solution (2 × 10 mL), dried over $MgSO_4$, filtered, concentrated

under vacuum to furnish the desired product as a white solid (148.4 mg, 89% yield, 99% HPLC purity).

Characterization

^{1}H NMR (400 MHz, $CDCl_3$): δ 7.37 – 7.28 (5H), 6.90 (br d, *J* = 6.8 Hz, 1H), 6.80 (br d, *J* = 8.4 Hz, 1H), 5.25 (br d, *J* = 8.3 Hz, 1H), 5.18 (d, *J* = 12.0 Hz, 1H), 5.12 (d, *J* = 12.4 Hz, 1H) 4.61 (qt, *J* = 7.2 Hz, 1H), 4.33 (t, *J* = 8.0 Hz, 1H), 3.94 (br t, *J* = 7.2 Hz, 1H), 2.09 (m, 1H), 1.94 – 1.82 (1H), 1.56 – 1.46 (1H), 1.42 (s, 9H), 1.38 (d, *J* = 7.2 Hz, 3H) 1.11 (m, 1H), 0.95 – 0.83 (12H).
^{13}C NMR (101 MHz, $CDCl_3$): δ 172.5, 172.0, 170.7, 156.1, 135.5, 128.7, 128.5, 128.3, 80.0, 67.2, 60.3, 57.8, 48.2, 37.0, 30.8, 28.4, 24.9, 19.4, 18.2, 18.1, 15.4, 11.3.
MS (ESI): m/z 492.1 $[M+H]^+$

TFA·H-Val-Ile-Ala-OBn

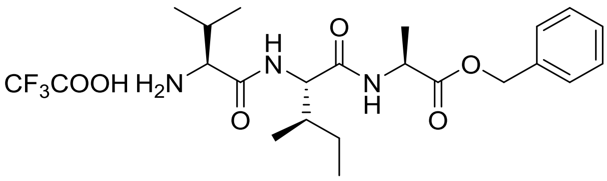

Synthesis in solution

Boc-Val-Ile-Ala-OBn (141.5 mg, 0.288 mmol, 1.0 equiv) was solubilized in a 1:1 TFA/CH_2Cl_2 mixture (1 mL) in a round-bottomed flask with magnetic stirring. After 90 min agitation, the reaction mixture was concentrated under vacuum to furnish the desired product as a yellow solid (146.8 mg, >99% yield, 92% HPLC purity).

Characterization

^{1}H NMR (400 MHz, CD_3OD): δ 7.45 – 7.28 (5H), 5.17 (d, *J* = 12.4 Hz, 1H), 5.13 (d, *J* = 12.0 Hz, 1H) 4.44 (q, *J* = 7.3 Hz, 1H), 4.27 (d, *J* = 8.0 Hz, 1H), 3.74 (d, *J* = 5.7 Hz, 1H), 2.17 (m, 1H), 1.81 (m, 1H), 1.59 (m, 1H), 1.39 (d, *J* = 7.3 Hz, 3H), 1.18 (m, 1H), 1.07 – 0.97 (m, 6H), 0.94 (d, *J* = 6.8 Hz, 3H), 0.88 (t, *J* = 7.4 Hz, 3H).
^{13}C NMR (101 MHz, CD_3OD): δ 173.6, 172.9, 169.5, 137.2, 129.6, 129.3, 67.9, 59.4, 59.2, 38.1, 31.7, 25.9, 18.9, 17.8, 17.3, 15.7, 11.3.
MS (ESI): m/z 392.3 $[M+H]^+$

HCl·H-Val-Ile-Ala-OBn

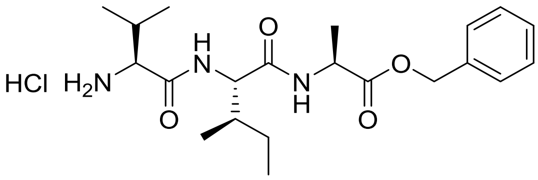

Synthesis in ball-mill

Boc-Val-Ile-Ala-OBn (121.2 mg, 0.247 mmol, 1.0 equiv) was submitted to gaseous HCl for 16 h. HCl·H-Val-Ile-Ala-OBn was recovered as a white solid (101.1 mg, 96% yield, 97% HPLC purity).

Characterization

^{1}H NMR (400 MHz, D_2O): δ 7.49 – 7.39 (5H), 5.21 (s, 2H), 4.43 (q, *J* = 7.3 Hz, 1H), 4.19 (d, *J* = 8.3 Hz, 1H), 3.85 (d, *J* = 6.1 Hz, 1H), 2.19 (m, 1H), 1.87 – 1.74 (1H), 1.55 – 1.46 (1H), 1.43 (d, *J* = 7.3 Hz, 3H), 1.17 (m, 1H), 1.02 – 0.98 (6H), 0.92 – 0.80 (6H).
^{13}C NMR (101 MHz, D_2O): δ 174.0, 172.6, 169.2, 135.2, 128.8, 128.7, 128.4, 67.7, 58.2, 48.9, 36.0, 30.1, 24.5, 17.6, 16.8, 16.5, 15.9, 14.5, 14.3, 10.0.
MS (ESI): m/z 392.3 $[M+H]^+$

Boc-Val-Val-Ile-Ala-OBn

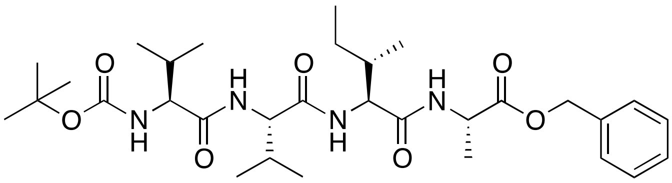

Synthesis in solution

TFA·H-Val-Ile-Ala-OBn (121.6 mg, 0.241 mmol, 1.0 equiv), Boc-Val-OH (62.8 mg, 0.289 mmol, 1.2 equiv), Oxyma (41.2 mg, 0.290 mmol, 1.2 equiv) and *N*,*N*-diisopropylethylamine (168 µL, 0.965 mmol, 4.0 equiv) were introduced in a round-bottomed flask and solubilized in a minimum amount of DMF (1.4 mL) with magnetic stirring. After a few minutes, *N*-(3-dimethylaminopropyl)-*N′*-ethylcarbodiimide (51.1 µL, 0.289 mmol, 1.2 equiv) was added. Aliquots of the reaction mixture were withdrawn periodically and immediately quenched with 1:1 MeCN/1 N aqueous HCl solution. The conversion was determined by HPLC analysis.

Table of conversion

Time (min)	0.0	5.0	10.0	20.0	40.0	60.0	120.0	180.0
Conversion in solution (%)	0.0	14.7	20.8	32.0	55.3	68.1	87.1	98.2

After stirring for 16 h, the reaction mixture was concentrated under vacuum and EtOAc (30 mL) and deionized water (25 mL) added. The aqueous phase was extracted with EtOAc (2 × 10 mL) and the combined organic phase was washed with 1 N aqueous HCl solution (2 × 10 mL), 1 N aqueous NaOH solution (2 × 10 mL) and saturated aqueous NaCl solution (2 × 10 mL), dried over $MgSO_4$, filtered and concentrated under vacuum to furnish the desired product as a white solid (91.1 mg, 64% yield, 85% HPLC purity).

Synthesis in ball-mill

HCl·H-Val-Ile-Ala-OBn (53.2 mg, 0.124 mmol, 1.0 equiv), Boc-Val-OH (32.5 mg, 0.150 mmol, 1.2 equiv), Oxyma (22 mg, 0.155 mmol, 1.2 equiv), NaH_2PO_4 (60.0 mg, 0.500 mmol,

4.0 equiv) and EtOAc (450 µL, η = 2.37 µL/mg) were introduced in a 15 mL PTFE grinding jar equipped with one stainless steel ball (10 mm diameter). The jar was subjected to grinding for 2 min in the mixer mill operated at 25 Hz. Then, *N*-(3-Dimethylaminopropyl)-*N′*-ethylcarbodiimide (26.4 µL, 0.149 mmol, 1.2 equiv) was added and the jar was subjected to grinding for 3 min in the mixer mill operated at 25 Hz. A first aliquot of the reaction mixture was withdrawn, immediately quenched with 1:1 MeCN/1 N aqueous HCl solution and analyzed by HPLC to determine the conversion. The jar was subjected to further grinding in the mixer mill operated at 25 Hz until next aliquot was withdrawn. This operation was repeated until completion of the reaction.

Table of conversion

Time (min)	0.0	5.0	10.0	20.0	30.0	40.0	60.0
Effective milling time (min)	0.0	3.0	6.0	14.0	22.0	30.0	50.0
Conversion in ball-mill (%)	0.0	72.4	87.2	100.0	100.0	100.0	100.0

To the reaction mixture was added EtOAc (30 mL) and deionized water (25 mL). The aqueous phase was extracted with EtOAc (2 × 10 mL) and the combined organic phase was washed with 1 N aqueous HCl solution (2 × 10 mL), 1 N aqueous NaOH solution (2 × 10 mL) and saturated aqueous NaCl solution (2 × 10 mL), dried over $MgSO_4$, filtered, concentrated under vacuum to furnish the desired product as a white solid (57.4 mg, 78% yield, 88% HPLC purity).

Characterization

^{1}H NMR (400 MHz, DMSO-*d*$_6$): δ 8.38 (d, *J* = 6.4 Hz, 1H), 7.80 (d, *J* = 8.8 Hz, 1H), 7.68 (d, *J* = 8.7 Hz, 1H), 7.41 – 7.28 (5H), 6.82 (d, *J* = 8.9 Hz, 1H), 5.09 (br s, 2H), 4.36 – 4.15 (3H), 3.78 (br t, *J* = 8.0 Hz, 1H), 2.00 – 1.84 (2H), 1.74 – 1.61 (1H), 1.47 – 1.32 (10H), 1.28 (d, *J* = 7.3 Hz, 3H), 1.05 (m, 1H), 0.90 – 0.69 (18H).
^{13}C NMR (101 MHz, DMSO-*d*$_6$): δ 172.2, 171.3, 170.7, 170.6, 155.5, 136.0, 128.4, 128.1, 128.0, 127.8, 78.0, 65.9, 60.1, 57.4, 56.3, 47.7, 36.7, 30.7, 30.1, 28.2, 24.2, 19.3, 19.2, 18.3, 18.1, 16.8, 15.0, 10.9.
MS (ESI): m/z 591.3 $[M+H]^+$

TFA·H-Val-Val-Ile-Ala-OH

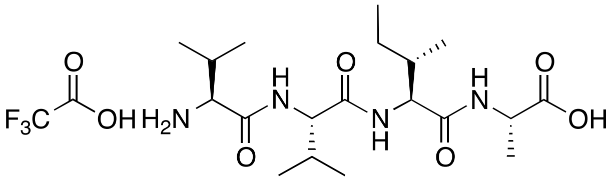

Solid-phase peptide synthesis

The peptide chains were elongated by means of a Liberty Blue peptide synthesizer (CEM) employing standard Fmoc chemistry. The syntheses were conducted on a 0.1 mmol scale starting with Fmoc-Ala-Wang resin (0.8 mmol/g) with a 5-fold excess of Fmoc-protected L-amino acids (0.2 M in DMF, 2.5 mL, 0.5 mmol, 5.0 equiv), 0.5 M DIC in DMF (1 mL, 0.5 mmol, 5.0 equiv) as coupling reagent, 1 M Oxyma in DMF (0.5 mL, 0.5 mmol, 5.0 equiv) for 7 min at 70 °C. The deprotection steps were carried out with piperidine/DMF 1:4 (v/v) (14 mL, 28.3 mmol, 283.5 equiv) over 3 min at 70 °C. The second coupling and deprotection of Fmoc-Ile-Ala-OHMP was performed during 90 min at room temperature. After the assembly was complete, the peptide-resin was washed with CH_2Cl_2 (70 mL) and cleavage was performed with TFA/TIS/H_2O 94:3:3 (10 mL) for 2 h at room temperature. The peptide was precipitated by adding cold Et_2O, the solution was decanted, and the solid was triturated with cold Et_2O, which was again decanted and filtrated. After lyophilization, the peptide was obtained as a fluffy white solid (28.0 mg, 54% yield, 96% purity, MS (ESI): m/z 401.3 $[M+H]^+$).

^{1}H and ^{13}C spectra

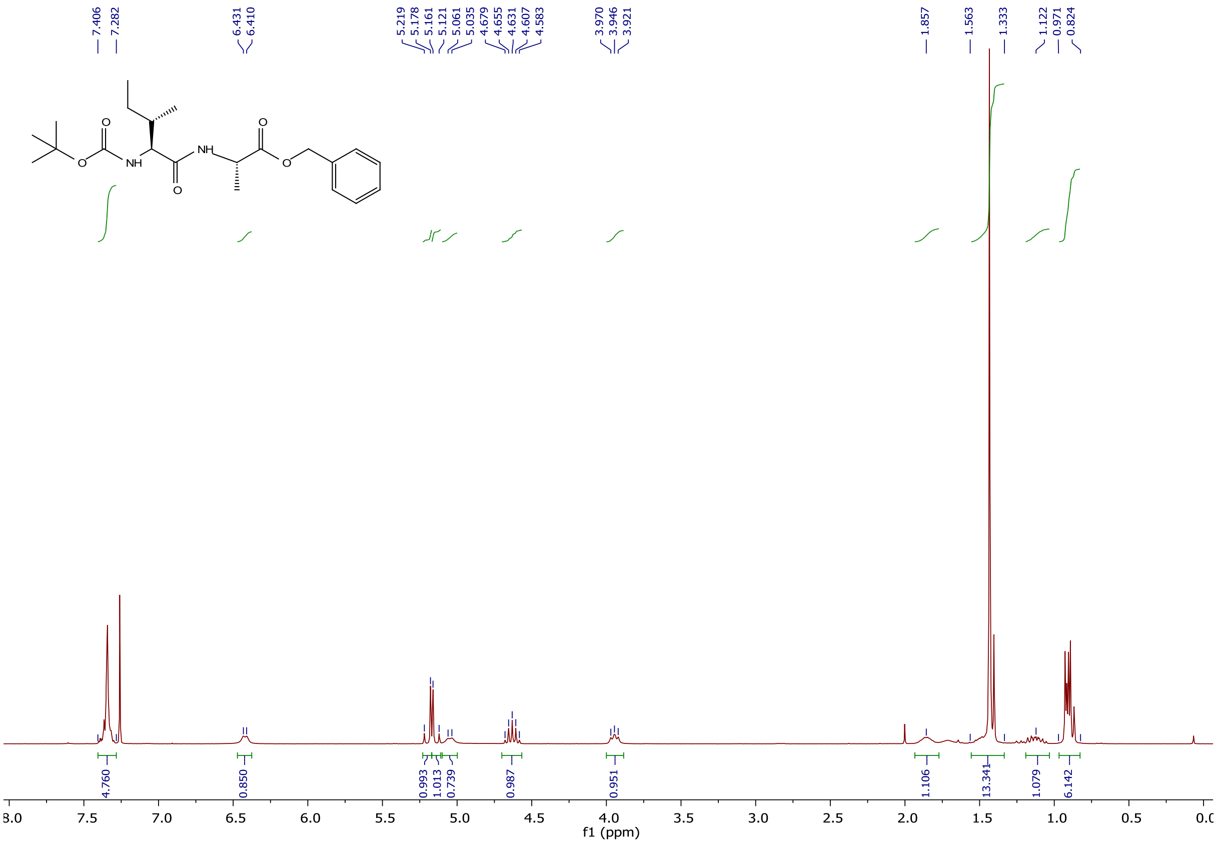

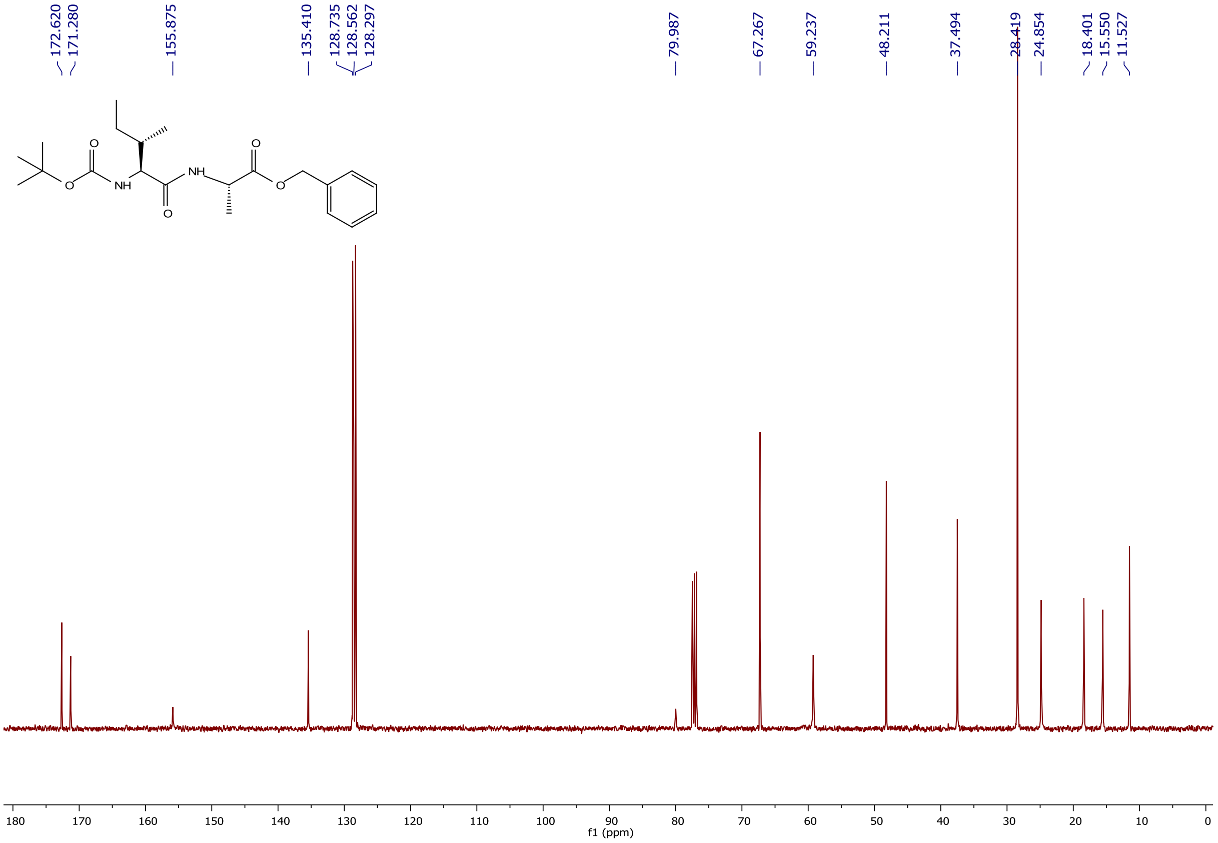

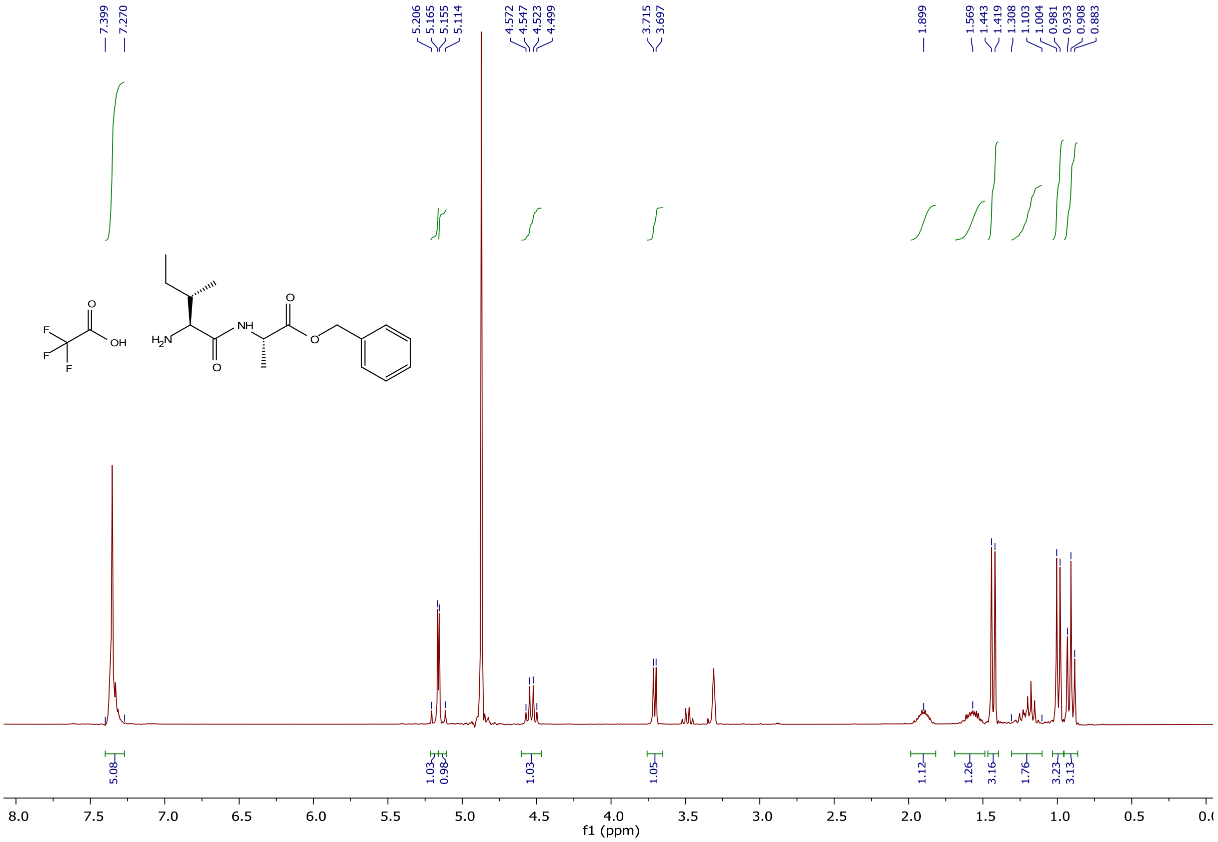

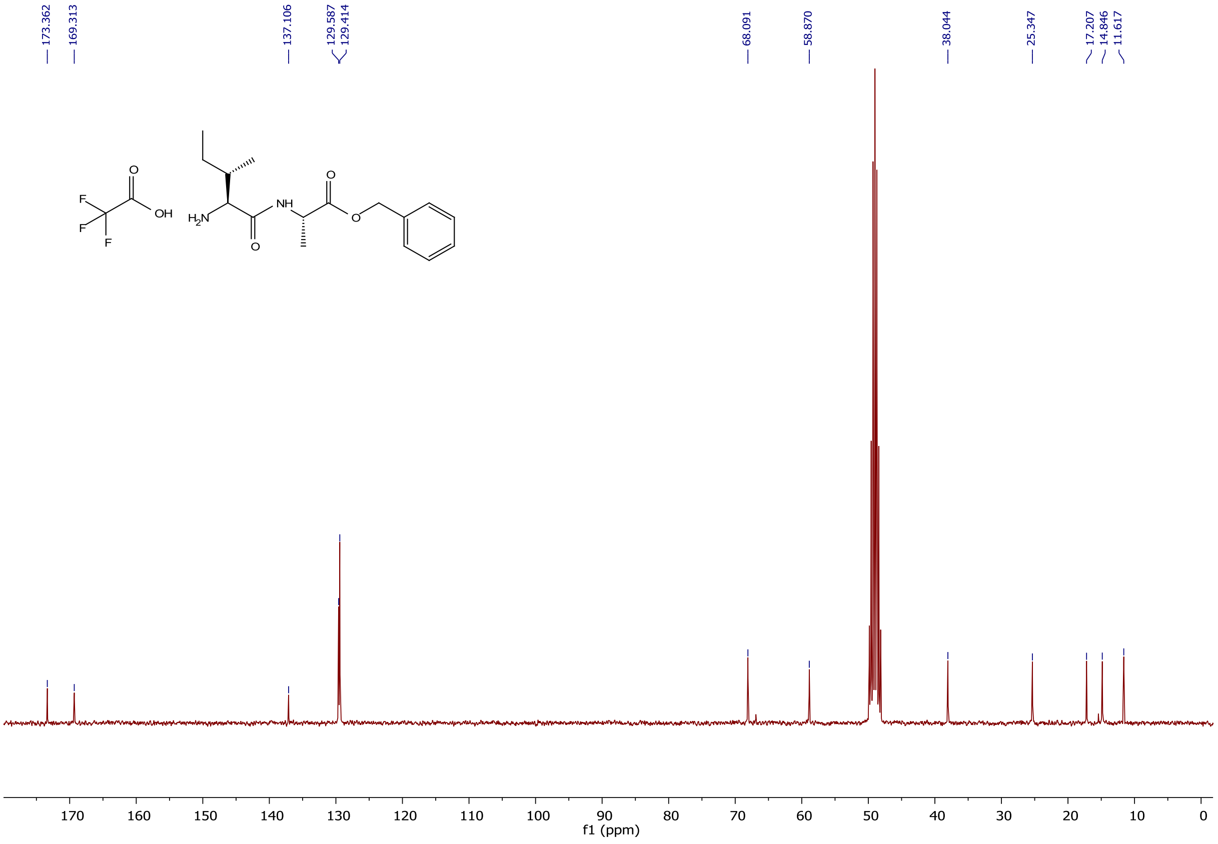

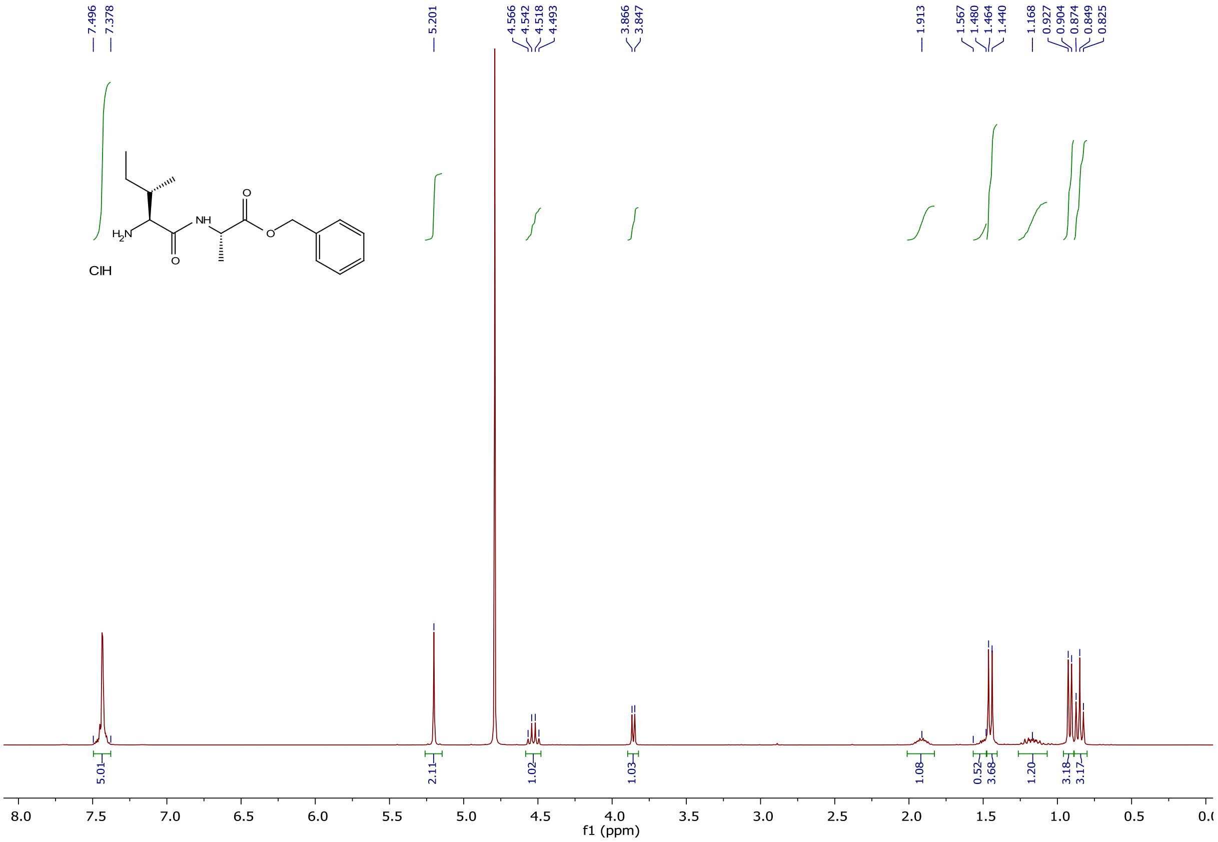
7.496
7.378
5.201
4.566
4.542
4.518
4.493
3.866
3.847
1.913
1.567
1.480
1.464
1.440
1.168
0.927
0.904
0.874
0.849
0.825
H2N
NH
O
O
O
ClH
5.01
2.11
1.02
1.03
1.08
0.52
3.68
1.20
3.18
3.17
f1 (ppm)

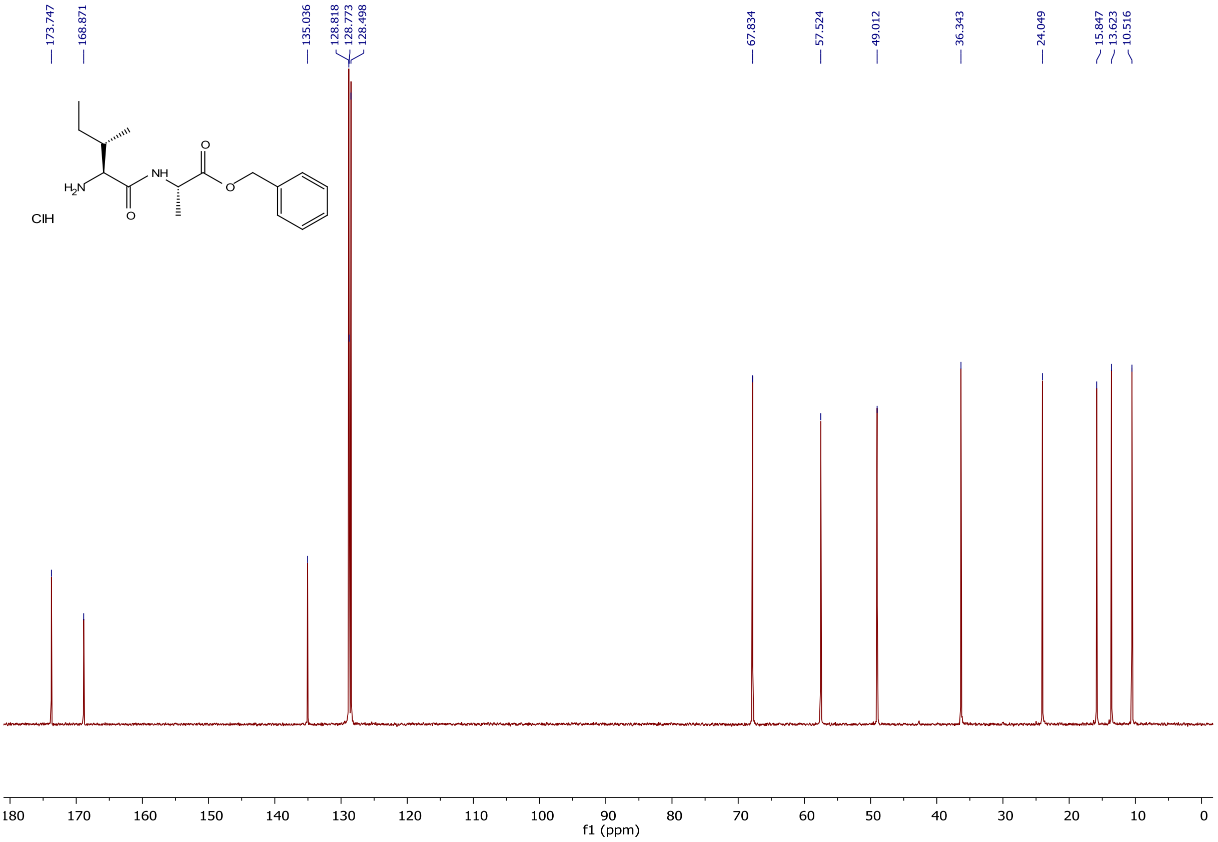
173.747
168.871
135.036
128.818
128.773
128.498
67.834
57.524
49.012
36.343
24.049
15.847
13.623
10.516
H2N
NH
O
O
O
ClH
f1 (ppm)

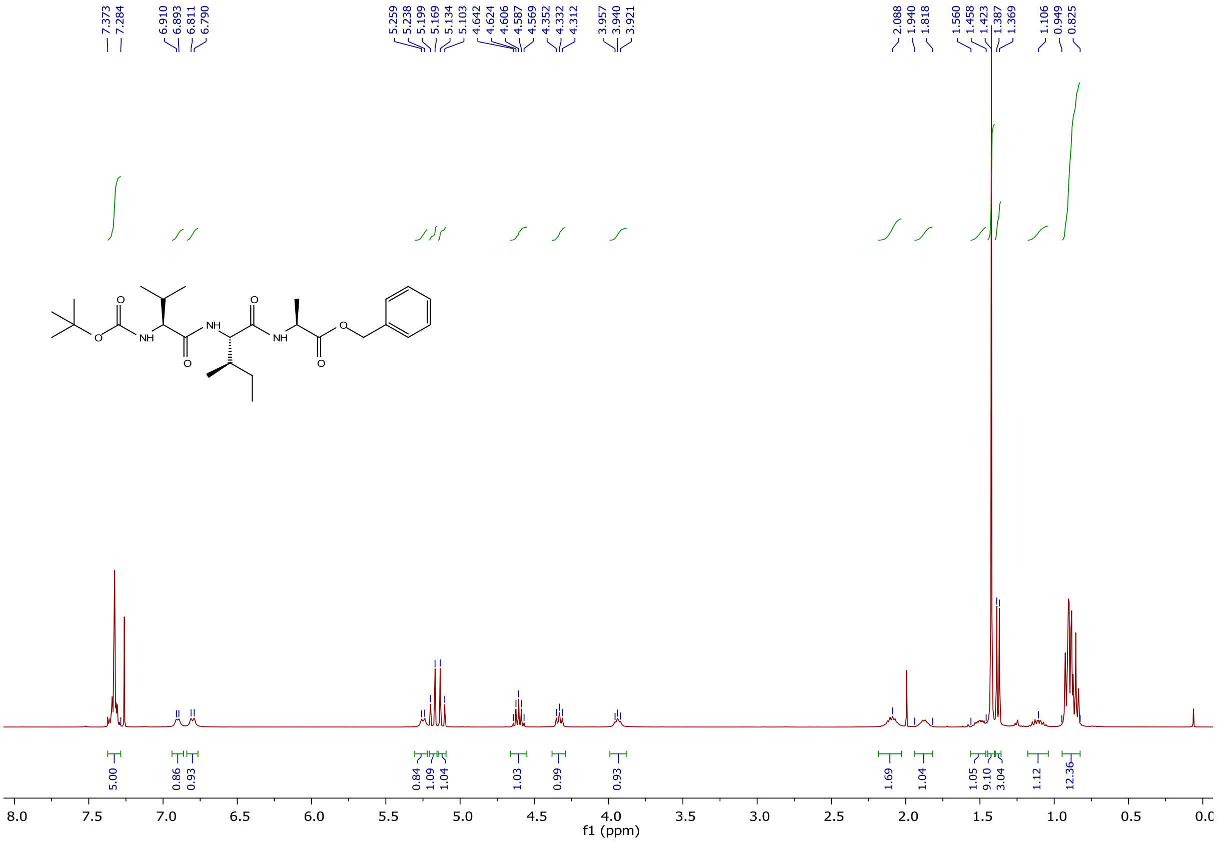
7.373
7.284
6.910
6.893
6.811
6.790
5.259
5.238
5.199
5.169
5.134
5.103
4.642
4.624
4.606
4.587
4.569
4.352
4.332
4.312
3.957
3.940
3.921
2.088
1.940
1.818
1.560
1.458
1.423
1.387
1.369
1.106
0.949
0.825
NH
NH
NH
O
O
O
O
O
O
5.00
0.86
0.93
0.84
1.09
1.04
1.03
0.99
0.93
1.69
1.04
1.05
9.10
3.04
1.12
12.36
8.0
7.5
7.0
6.5
6.0
5.5
5.0
4.5
4.0
3.5
3.0
2.5
2.0
1.5
1.0
0.5
f1 (ppm)

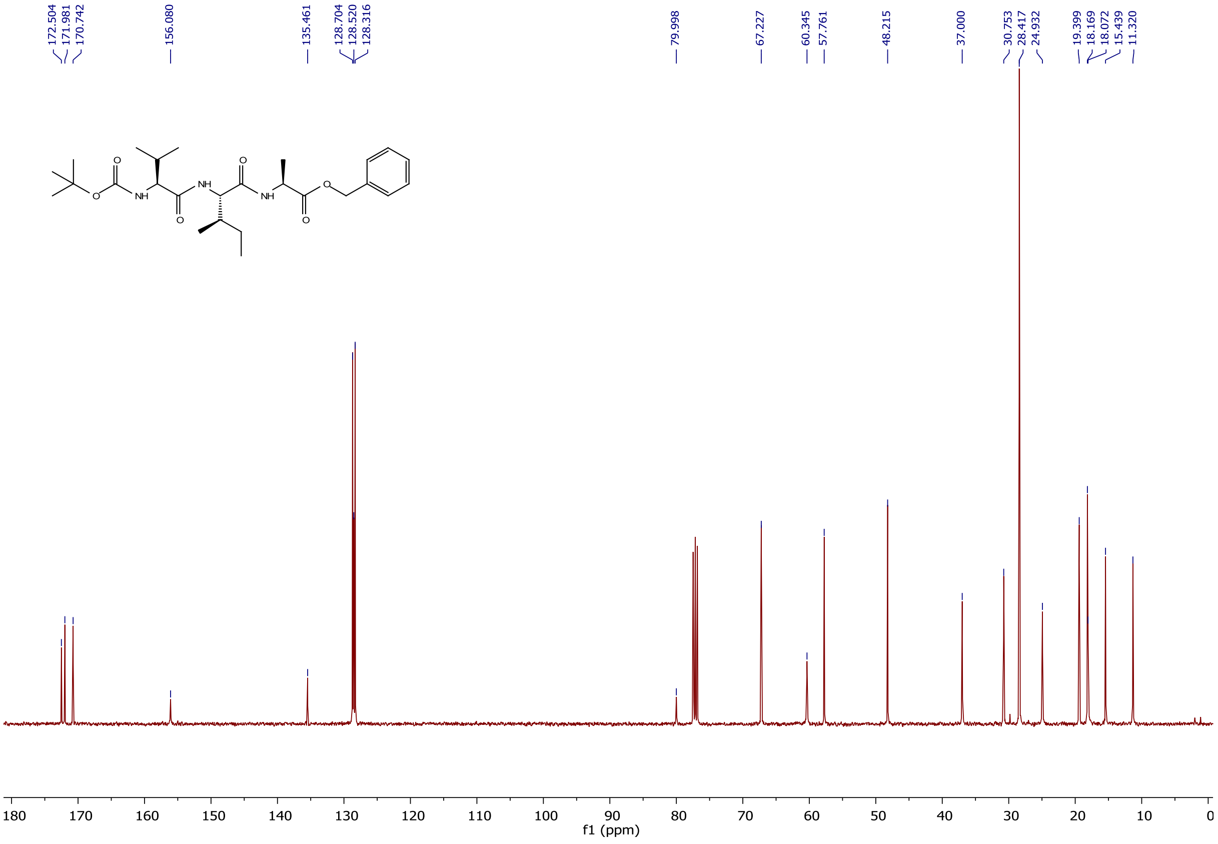
172.504
171.981
170.742
156.080
135.461
128.704
128.520
128.316
79.998
67.227
60.345
57.761
48.215
37.000
30.753
28.417
24.932
19.399
18.169
18.072
15.439
11.320
NH
NH
NH
O
O
O
O
O
O
180
170
160
150
140
130
120
110
100
90
80
70
60
50
40
30
20
10
0
f1 (ppm)

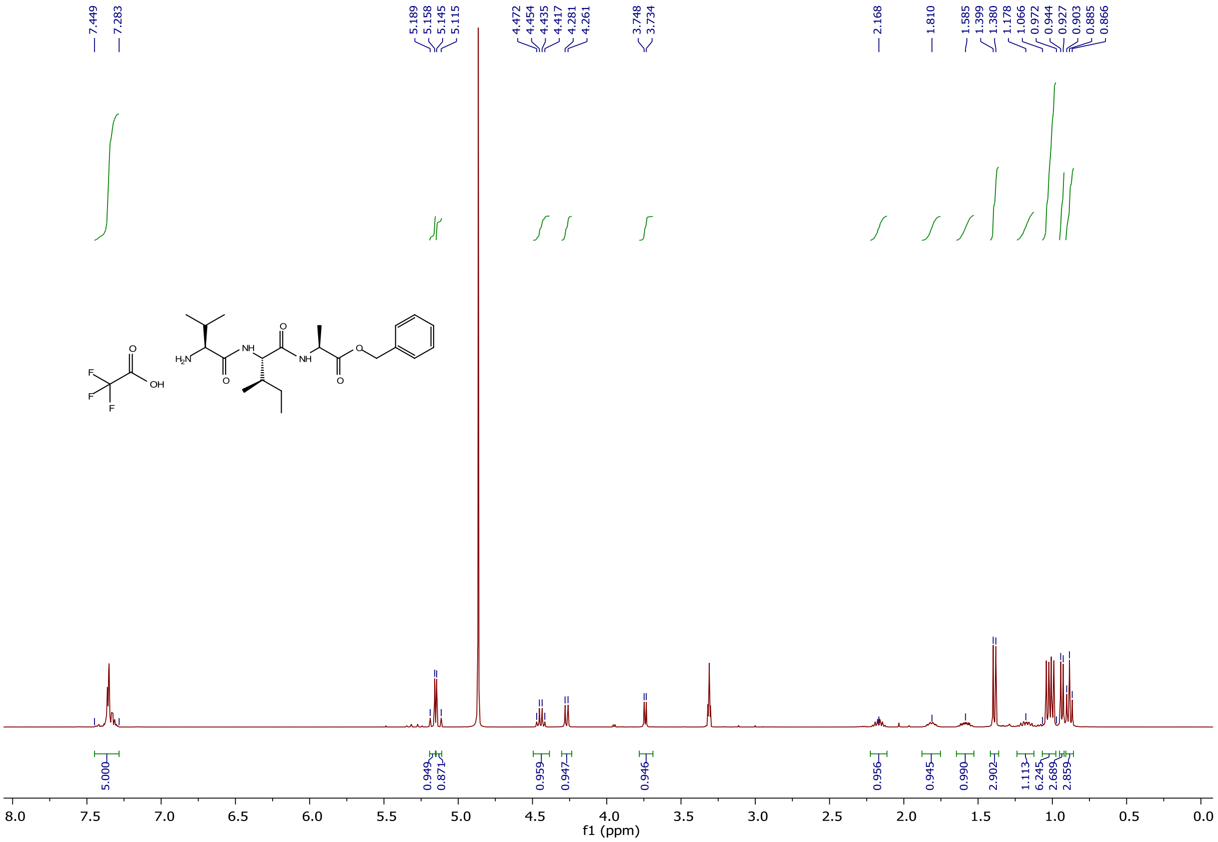
7.449
7.283
5.189
5.158
5.145
5.115
4.472
4.454
4.435
4.417
4.281
4.261
3.748
3.734
2.168
1.810
1.585
1.399
1.380
1.178
1.066
0.972
0.944
0.927
0.903
0.885
0.866
5.000
0.949
0.871
0.959
0.947
0.946
0.956
0.945
0.990
2.902
1.113
6.245
2.689
2.859
f1 (ppm)

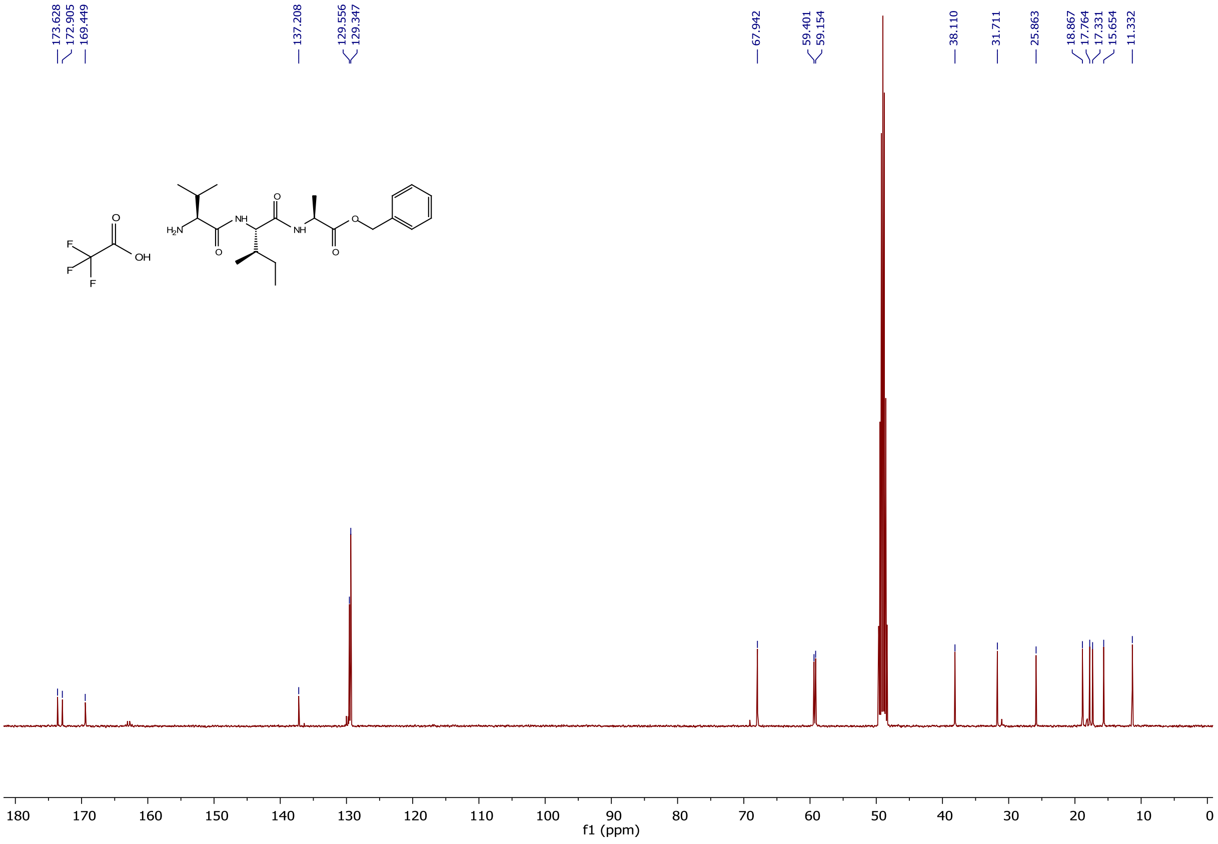
173.628
172.905
169.449
137.208
129.556
129.347
67.942
59.401
59.154
38.110
31.711
25.863
18.867
17.764
17.331
15.654
11.332
f1 (ppm)

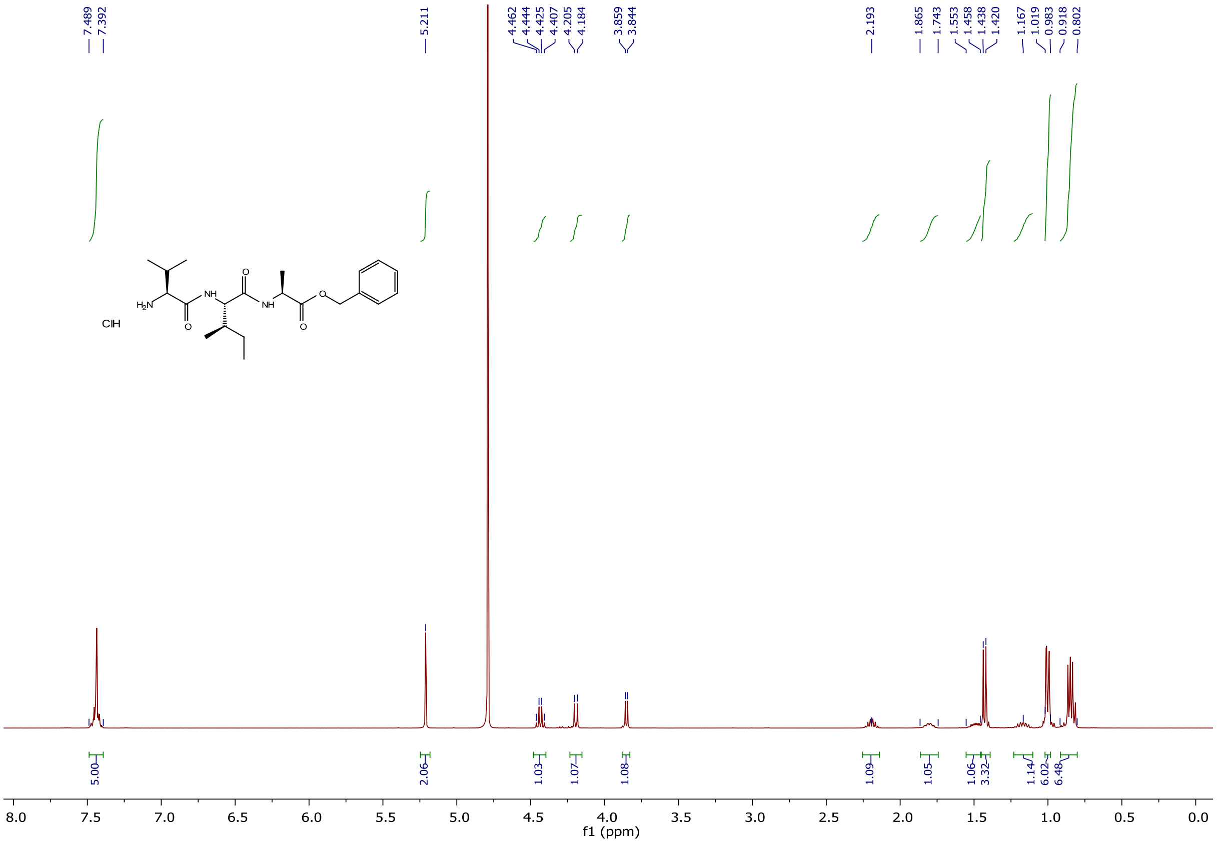
7.489
7.392
5.211
4.462
4.444
4.425
4.407
4.205
4.184
3.859
3.844
2.193
1.865
1.743
1.553
1.458
1.438
1.420
1.167
1.019
0.983
0.918
0.802
H2N
NH
NH
O
O
O
O
ClH
5.00
2.06
1.03
1.07
1.08
1.09
1.05
1.06
3.32
1.14
6.02
6.48
f1 (ppm)

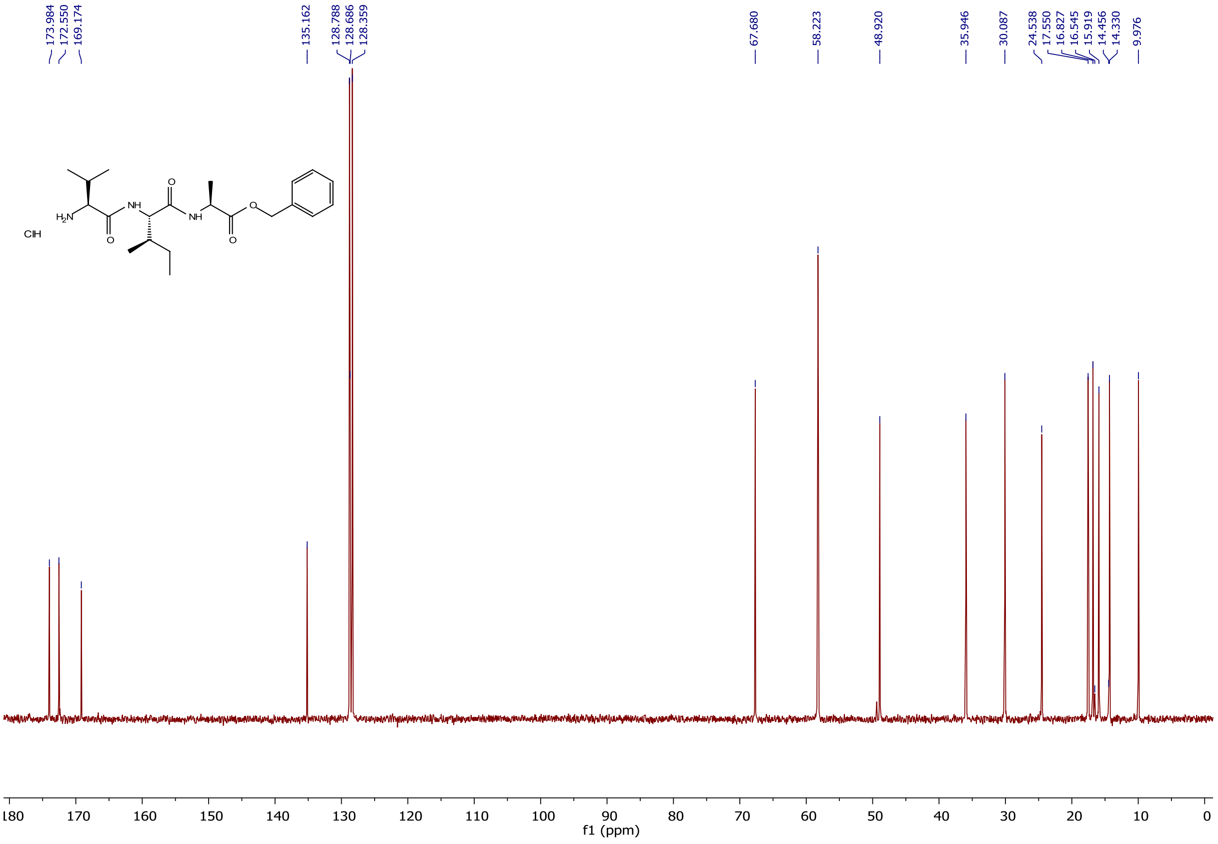
173.984
172.550
169.174
135.162
128.788
128.686
128.359
67.680
58.223
48.920
35.946
30.087
24.538
17.550
16.827
16.545
15.919
14.456
14.330
9.976
H2N
NH
NH
O
O
O
O
ClH
f1 (ppm)

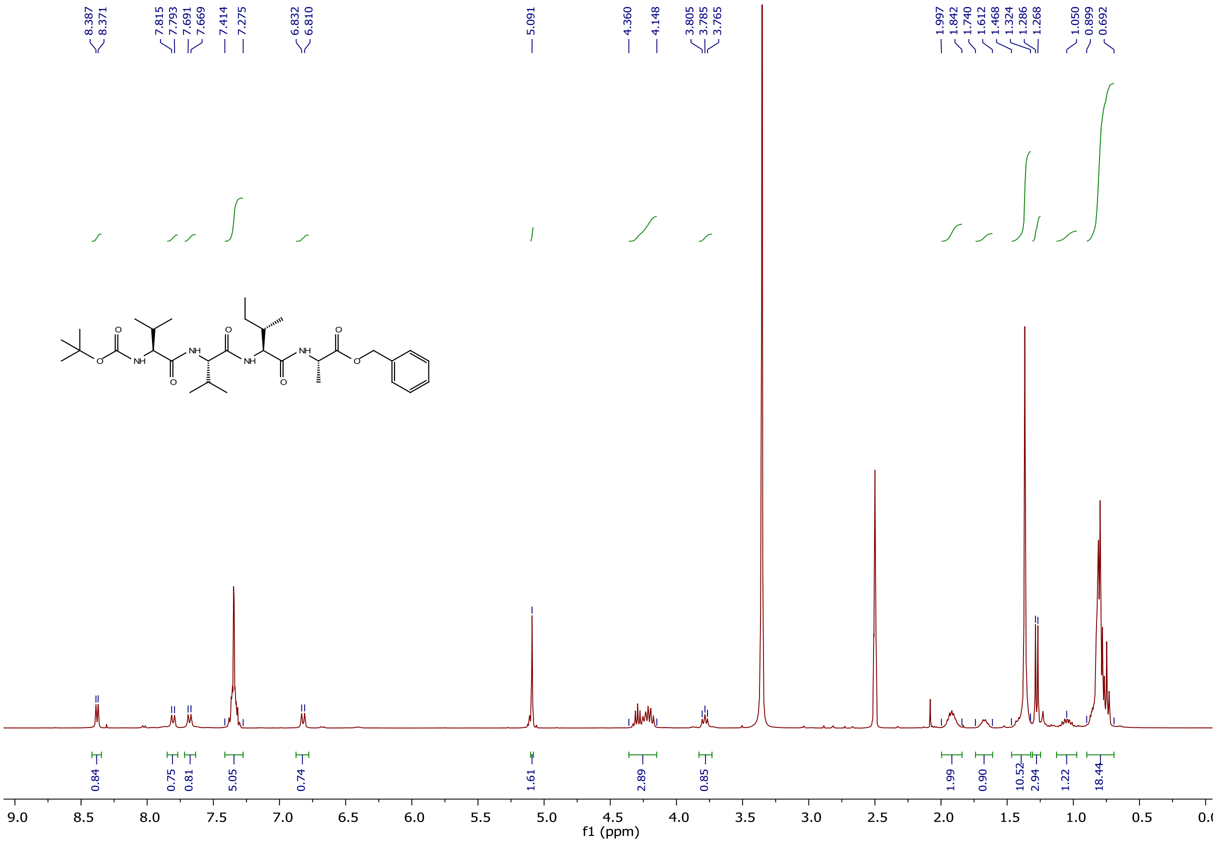
8.387
8.371
7.815
7.793
7.691
7.669
7.414
7.275
6.832
6.810
5.091
4.360
4.148
3.805
3.785
3.765
1.997
1.842
1.740
1.612
1.468
1.324
1.286
1.268
1.050
0.899
0.692
0.84
0.75
0.81
5.05
0.74
1.61
2.89
0.85
1.99
0.90
10.52
2.94
1.22
18.44
f1 (ppm)

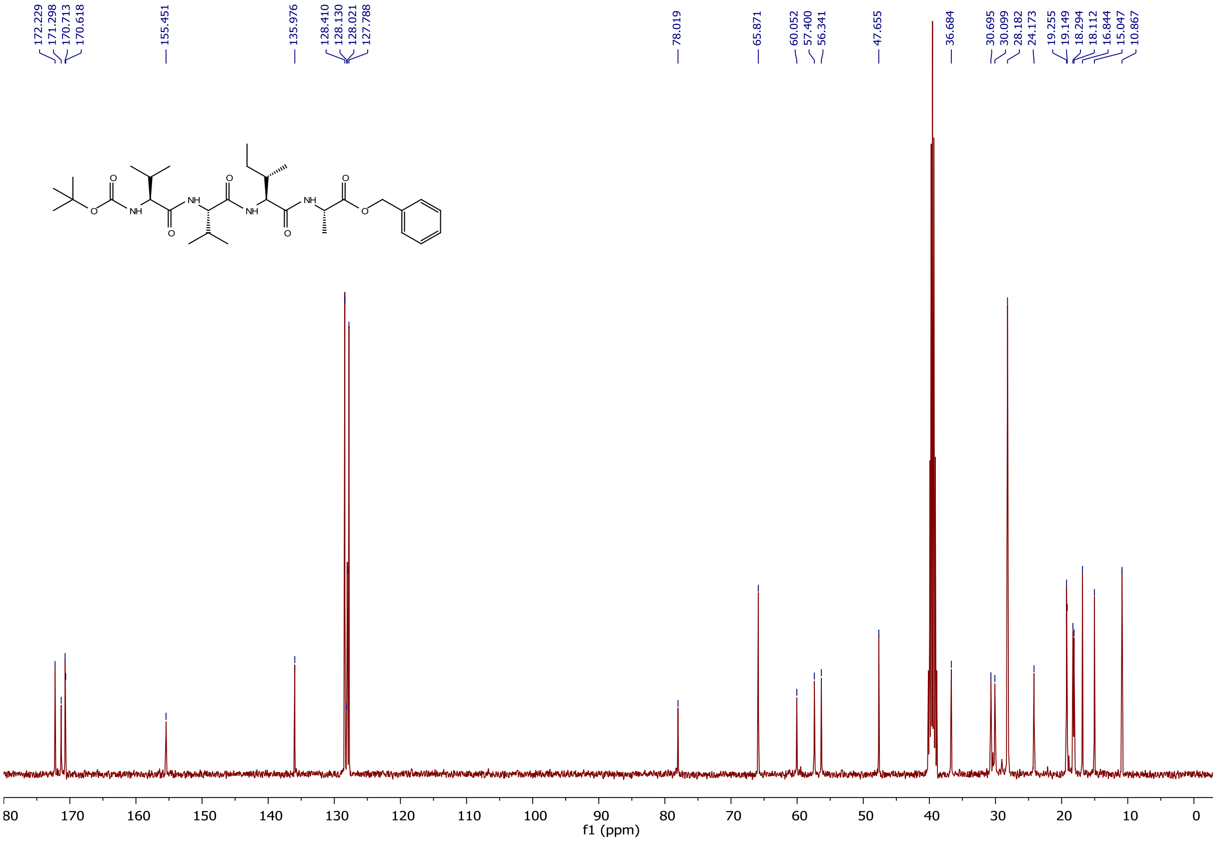
172.229
171.298
170.713
170.618
155.451
135.976
128.410
128.130
128.021
127.788
78.019
65.871
60.052
57.400
56.341
47.655
36.684
30.695
30.099
28.182
24.173
19.255
19.149
18.294
18.112
16.844
15.047
10.867
f1 (ppm)

References

(1) Fustero, S.; Sancho, A. G.; Chiva, G.; Sanz-Cervera, J. F.; del Pozo, C.; Aceña, J. L. *J. Org. Chem.* **2006**, *71*, 3299-3302.
(2) Sho-kichi, O.-u.; Jin-Shik, L.; Mitsuaki, N. *Bull. Chem. Soc. Jpn.* **1996**, *69*, 1303-1307.
